# Investigation of the Initial Corrosion Destruction of a Metal Matrix around Different Non-Metallic Inclusions on Surfaces of Pipeline Steels

**DOI:** 10.3390/ma15072530

**Published:** 2022-03-30

**Authors:** Elena Sidorova, Andrey Karasev, Denis Kuznetsov, Pär G. Jönsson

**Affiliations:** 1 Department of Materials Science and Engineering, KTH Royal Institute of Technology, Brinellvägen 23, 100 44 Stockholm, Sweden; parj@kth.se; 2 Department of Functional Nanosystems and Hightemperature Materials, National University of Science and Technology (MISIS), Leninsky Prospect 4, 119049 Moscow, Russia; dk@misis.ru

**Keywords:** pipeline steels, non-metallic inclusions, corrosion, electrolytic extraction, chemical extraction

## Abstract

Typical non-metallic inclusions in two industrial low-carbon steels for oil pipelines were investigated as three-dimensional objects on film filters after electrolytic extraction and filtration of metal samples. A method of soft chemical extraction using a 10%AA electrolyte was used to study the initial corrosion process in the steel matrix surrounding various non-metallic inclusions. To determine and compare “corrosive” inclusions and their influence on the initial stages of corrosion of the adjacent layer of the steel matrix, quantitative parameters (such as the diameter of the corrosion crater (*D_cr_*) and pit (*D_pit_*), and the relative dissolution coefficient of the metal matrix (*KD*) around various inclusions) were determined after chemical extraction. It was found that CaO-Al_2_O_3_-MgO oxides and TiN inclusions did not cause an initial corrosion of the steel matrix surrounding these inclusions. However, tensile stresses in the steel matrix occurred around CaS inclusions (or complex inclusions containing a CaS phase), which contributed to the initiation of corrosion around these inclusions.

## 1. Introduction

In connection with progress in the world of energy and oil production, the issue of ensuring the corrosion resistance of pipe materials is becoming more and more urgent.

Moreover, the development of new mineral deposits, as well as the tightening of environmental controls, highlights the importance of an improved understanding of corrosion. In modern steels used for transporting oil and gas, alloying and microalloying elements are added to increase corrosion resistance [1,2]. The influence of these elements on corrosion has been widely studied [1,3,4,5]. In addition to the importance of the chemical composition of steel, it has been found that the microstructure plays an important role in the corrosion process, especially with respect to local corrosion of pipeline steels [6,7,8,9,10]. Moreover, most researchers believe that inclusions in steels act as initiators of corrosion pits [11,12,13].

Non-metallic inclusions (NMIs) are compound materials formed within steel during the manufacturing process. Inclusions can have different chemical and technological origins and can influence various mechanical and physical properties of steels. These include plastic characteristics, fatigue strength, corrosion resistance, wear resistance, etc. It has been established that the initiation of cracks during SCC (stress corrosion crack) formation and HIC (hydrogen crack) formation in pipeline steels is caused by uneven structures and microstructures, as well as by non-metallic inclusions [7,14,15]. Therefore, in the study of the corrosion process, much attention is paid to the mechanism of the initiating effect of inclusions on the pitting corrosion in the steel matrix. More specifically, it has been found that the chemical dissolution of complex inclusions, or parts of them, induces corrosion pits [16]. Some authors [16,17,18,19] have found that sulfide-containing inclusions are corrosion initiators in the process of chemical etching of the metal matrix surrounding inclusions. Complex inclusions of calcium aluminate sludge with calcium sulfide shells are also unfavorable in terms of local corrosion damage [20]. Other researchers have confirmed that microdefects, which arise due to different coefficients of thermal expansion, and to contrasting mechanical properties between the inclusion and the metal matrix, cause local corrosion around such inclusions [21].

For production of economically viable, low-alloy pipeline steels, an important first stage is the deoxidation of steel, which is commonly performed using aluminum [22]. In this regard, various types of inclusion, such as pure aluminum oxides (Al_2_O_3_) and various types of spinel (mainly MgO-Al_2_O_3_), are found in these steels [19]. In the case of Ca treatment of liquid steels in the ladle before casting, modified inclusions of calcium aluminates (*x*CaO-*y*Al_2_O_3_) and calcium sulfides (CaS) can be detected in the metal [23]. In [24], in similar low-carbon steels, the authors found more complex inclusions consisting of a composite type containing three or more phases. Therefore, it is important to understand the main mechanisms underlying the emergence and development of pitting corrosion of pipeline steels containing different non-metallic inclusions, as well as what factors influence these inclusions and how their characteristics affect the corrosion process.

It is known that local corrosion is a consequence of the effect of the electrolyte solution on the inclusion and/or the metal matrix around the inclusion. The dissolution of one of these components inevitably leads to the formation of micro-crevices in the area, and subsequently to pitting zones of pitting corrosion. The phenomenon of the dissolution of inclusions and metal matrix zones around them for pipeline steels has been extensively studied in order to establish the main mechanisms of the process. Table 1 lists published studies concerning localized corrosion induced by different complex inclusions in low-alloy steels [7,9,17,18,22,25,26]. These studies provide a broad analysis of the initiation mechanisms of localized corrosion in aqueous corrosion solutions. The research cited has concentrated on qualitative study of the morphology and composition of inclusions and their direct influence during exposure to different experimental electrolyte solutions. However, in the studies listed, there has been no quantitative analysis of non-metallic inclusions to assess the nature of their influence on the process of the initiation of corrosion, or in relation to identifying the most harmful inclusions.

In previous work [27], we focused on investigation of the factors in metallurgical production that affect modification of various non-metallic inclusions during the smelting of low-carbon pipeline steel. Since the chemical composition and microstructure, consisting of a mixture of pearlite and ferrite, in two investigated pipeline steels were very similar, the observed difference in corrosion resistance of these steels was attributed to the effect of the presence of various non-metallic inclusions.

Non-metallic inclusions and their characteristics (such as composition, morphology and quantity) were studied using three-dimensional investigation of the NMIs after electrolytic extraction from steel samples. Based on the obtained results, the most corrosive inclusion types were determined.

This study focused on the investigation and quantitative evaluation of the initial stage of local corrosion destruction of the metal matrix surrounding different non-metallic inclusions present on the surface of pipeline steels. Based on the technique applied and the results obtained, more harmful, corrosion-active non-metallic inclusions, causing corrosion dissolution of the metal matrix, were identified for pipeline steels.

## 2. Experimental Procedures

### 2.1. Materials and Procedures

Two heats (Steel A and Steel B) of industrial low-carbon steel used for production of oil-pipelines were used in this study. Both steels were obtained using the same production process, which included the following stages: converter, ladle treatment, DH-vacuum treatment, and continuous casting, followed by hot rolling of slabs into sheets of up to 8 mm thickness. For modification of the non-metallic inclusions in the steel melt, 0.25 kg/t (in Steel A) and 0.23 kg/t (in Steel B) of calcium carbide were added during the DH-vacuum treatment. Details of the steelmaking process of these heats are given in a previous publication [27].

The steel sheets were produced using hot rolling at mill 2000 followed by laminar cooling. Steel samples for estimation of the initial corrosion destruction of the metal matrix around different types of non-metallic inclusions were cut from the sheets of each heat. Their chemical compositions are given in Table 2 (marked as Steel A representing a conventional production heat and Steel B representing an experimental production heat). The samples had a similar content of carbon and alloying elements (such as chromium, nickel, copper, titanium, niobium and vanadium). However, the Al, Ca, and S content in Steel A was larger and the N content was lower in comparison to Steel B. Both steels had similar yield strength (600 and 560 MPa for Steels A and B, respectively) and the same tensile strength (500 MPa).

### 2.2. Extractions and Investigations of Inclusions

For the three-dimensional (3-D) investigation and characterization of non-metallic inclusions in the given steels, NMIs were electrolytically extracted from steel samples using 10%AA electrolyte (10% acetylacetone–1% tetramethyl-ammonium chloride-methanol). The electrolytic extraction method was based on the fast electrolytic dissolution of the steel matrix of the samples using a soft electrolyte so as not to cause the dissolution of non-metallic inclusions. The following electric parameters were used in the EE process: electric currents of 40–60 mA and voltages of 2.9–3.8 V. After extraction of inclusions from steel samples, the electrolyte containing undissolved NMIs was filtered through a polycarbonate film filter with a hole diameter of 0.4 μm. The weight of the steel samples dissolved during EE varied between 0.21 and 0.24 g, while the depth of the dissolved metal layer varied between 0.18 and 0.32 mm.

**Figure 1:**
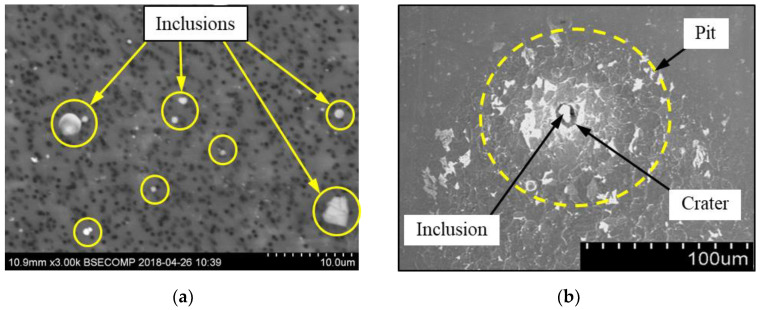
Typical SEM images of non-metallic inclusions observed on a film filter (**a**) and a metal surface (**b**) after EE.

The extracted inclusions were investigated as 3-D objects on a surface of film filter using a scanning electron microscope (SEM) equipped with energy dispersive spectroscopy (EDS). Using this method, the main characteristics, such as the size, number, composition and morphology, of the extracted non-metallic inclusions could be determined more precisely compared to conventional two-dimensional (2-D) investigation of sliced NMIs on a polished surface of a metal sample. Typical SEM images of different inclusions observed on a film filter and metal surface after EE are shown in Figure 1.

The composition of the main type of inclusions and their phases was determined based on EDS measurement of 15 to 25 inclusions for each type of investigated NMI. For an evaluation of the inclusion number per unit volume of steel sample (*N*_V_), more than 200 inclusions were measured in each sample. The values of *N*_V_ for each size interval were calculated using the following equation [28]:

(1) $$N_{V}=n\cdot \frac{A_{f}}{A_{obs}}\cdot \frac{\rho_{m}}{W_{dis}}$$

where *n* is the number of measured inclusions in the given size interval; *ρ_m_* is the density of the steel samples (~0.0078 g/mm^3^); *A_f_* and *A_obs_* are the total area of the film filter containing extracted inclusions after filtration (~1200 mm^2^) and the area of filter observed by SEM, respectively; *W_dis_* is the weight of the steel dissolved during the electrolytic extraction.

The equivalent size (*d_eq_*) for each inclusion observed on SEM images was determined as the average value of the measured maximum length (*L*) and width (*W*):

(2) $$d_{eq}=\frac{\;\left(L+W\right)}{2}$$

For an evaluation of the initial stages of local corrosion destruction of metal matrix around different non-metallic inclusions on the surface of the pipeline steels, the polished steel samples were held for 30 min in 10%AA electrolyte without applying an electric current. After that, the inclusions and the dissolved metal matrix around them were investigated on sample surfaces using SEM in combination with EDS. A typical SEM image of the dissolution of the metal matrix around an inclusion on a sample surface is shown in Figure 1b. Inoue et al. [28] reported that 10%AA electrolyte can be used for the extraction of most inclusions (including Al_2_O_3_, CaO-Al_2_O_3_, CaO-SiO_2_, TiN, CaS and others) without dissolving the inclusions during the EE process. Therefore, it was assumed that the inclusions were not directly involved in the dissolution process of the metal matrix around the inclusion due to chemical reaction with the electrolyte. This method of chemical dissolution of steel samples in the electrolyte is denoted “chemical extraction” (ChE) in this study.

After ChE, the areas of inclusions (*A_inc__l_*), deep corrosion craters (*A_cr_*) and wide corrosion pits (*A_pit_*) present around the investigated inclusions on metal surfaces were measured using image analysis software. The calculated diameters of corrosion craters (*D_cr_*) and pits (*D_pit_*) and the relative coefficients of metal matrix dissolution (*KD*) around different inclusions were used for a quantitative evaluation of the influence of inclusions on the corrosion destruction of a steel matrix. The values of *D_cr_*, *D_pit_* and *KD* were calculated as follows:

(3a) $$D_{cr}=\sqrt{\left(4\cdot A_{cr}/\pi \right)}$$

(3b) $$D_{pit}=\sqrt{\left(4\cdot A_{pit}/\pi \right)}$$

(4a) $$KD_{cr}=A_{cr}\;/A_{incl}$$

(4b) $$KD_{pit}=A_{pit}\;/A_{incl}$$

## 3. Results and Discussions

### 3.1. Characterization of Typical Non-Metallic Inclusions after Electrolytic Extraction of Steel Samples

Based on the results of investigation of non-metallic inclusions on film filters after electrolytic extraction, and on surfaces of metal samples after chemical extraction, the observed NMIs were classified in the following four groups: (i) Group 1 containing pure CaS inclusions and oxide cores completely covered by a CaS layer (with a concentration of CaS in the outer inclusion layer reaching 97–100%); (ii) Group 2 containing CaO-Al_2_O_3_-MgO inclusions partially covered by CaS; (iii) Group 3 containing oxide inclusions which have precipitated on inclusion surfaces of CaS and TiN phases, and (iv) Group 4 containing oxide inclusions completely covered by TiN and pure TiN inclusions (the concentration of TiN in the outer inclusion layer reaching 78–100%). Some oxide inclusions can contain up to 7% of SiO_2_. The SEM images, composition and size ranges of these typical inclusions are given in Table 3 in which the following abbreviations are used for the oxide components: A—Al_2_O_3_; M—MgO; Si—SiO_2_; C—CaO.

**Figure 2:**
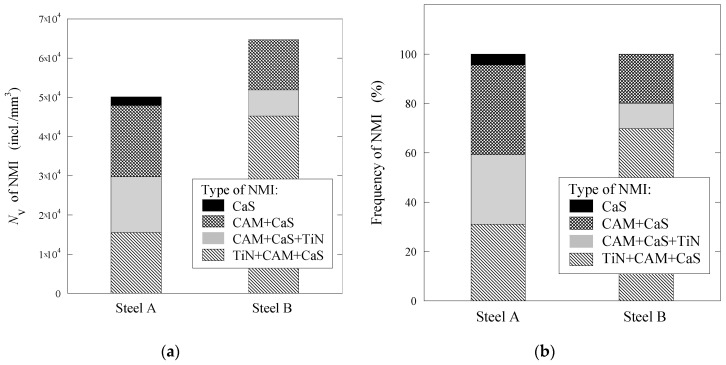
Number of inclusions per unit volume (**a**) and frequencies (**b**) of different types of non-metallic inclusions observed on film filters after electrolytic extraction of Steels A and B.

The number of observed inclusions per unit volume, which was calculated using Equation (1), and the frequencies of different non-metallic inclusions observed on film filter after electrolytic extraction of both steels are given in Figure 2. The number of inclusions in steel B was approximately 23% larger compared to Steel A. This can be explained by the presence of a larger quantity of small inclusions (<2 µm) in Steel B, as was described in a previous publication [27]. However, Steel A contained significantly larger numbers of inclusions containing CaS (3.5% CaS, 37.0% CAM + CaS and 28.5% CAM + CaS + TiN) compared to Steel B (20% CAM + CaS and 10% CAM + CaS + TiN). However, Steel B contained up to 70% of inclusions with a high concentration of TiN inclusions (Group 4), but no pure CaS inclusions (Group 1).

### 3.2. Evaluation of Depletion Zone of Steel Matrix around Different NMIs

**Figure 3:**
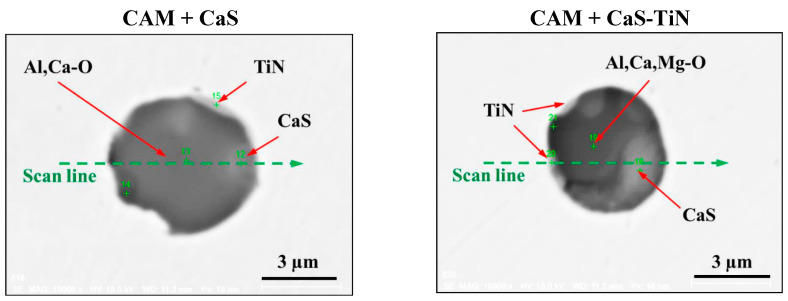

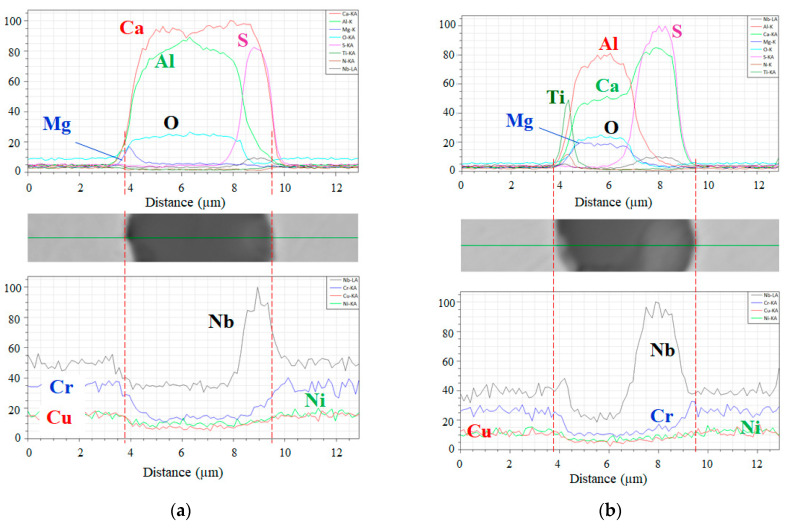
Distribution of main components present in inclusions and the main alloying elements present in the steel matrix around typical NMIs.

It is well known that one of the possible reasons for corrosion of steel around some non-metallic inclusions is a depleted zone of the steel matrix around these inclusions. Here, the concentration of anticorrosion alloying elements was significantly reduced due to the involvement of these elements in the formation of the inclusions. Therefore, a preliminary evaluation of heterogeneity (depletion zones) of the steel matrix around different NMIs using EDS scanning of the metal matrix and inclusions was performed on polished metal surfaces of both steel samples. The distribution of the main components in inclusions and the main alloying elements in the steel matrix around typical NMIs of Groups 2 and 3 are shown in Figure 3. The contents of the main alloying elements in the steel matrix around NMI were almost constant. Therefore, a perceptible depletion zone of the main alloying elements (such as Cr, Ni, Cu and Nb) was not observed in the steel matrix around non-metallic inclusions belonging to Groups 2 and 3. A similar absence of depletion in the steel matrix was observed around inclusions belonging to Groups 1 and 4.

Investigation of NMIs on the surfaces of polished steel samples revealed small voids (or micro-crevices) around some inclusions. However, the size of most observed voids located close to these non-metallic inclusions was less than 0.3 µm.

### 3.3. Evaluation of Corrosion Destruction of a Steel Matrix around Different NMI

**Figure 4:**
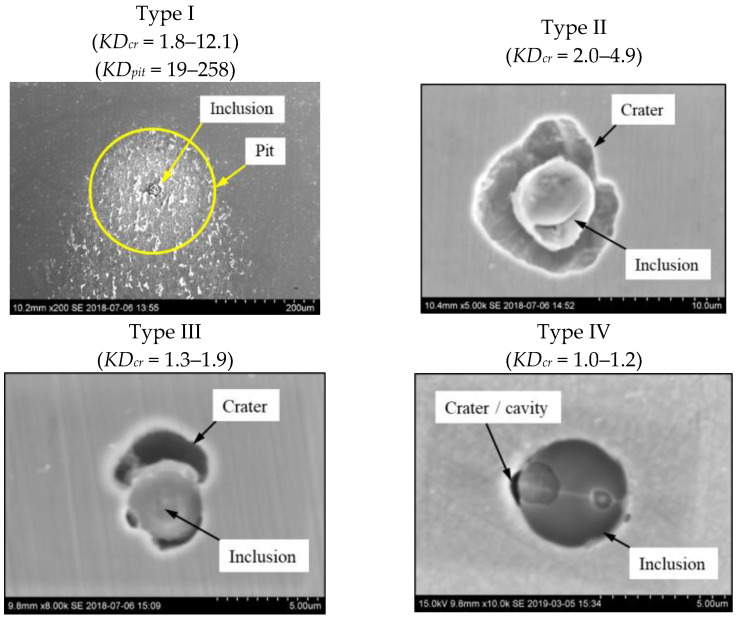
Different types of corrosion dissolution of the metal matrix around various non-metallic inclusions.

According to results obtained by investigation of the initial corrosion dissolution of a metal matrix around different NMIs on surfaces of steel samples after 30 min of chemical extraction in the 10%AA electrolyte, all inclusions were divided into four types: (i) Type I included NMIs connected to a corrosion crater (*KD_cr_* = 1.8–12.1) as well as a visual wide corrosion dissolution of metal matrix surface around these inclusions, denoted in this study as “pit” (*KD_pit_* = 19–258); (ii) Type II inclusions were NMIs having deep corrosion craters (*KD_cr_* = 2.0–4.9) without having a wide corrosion dissolution of the metal matrix surface; (iii) Type III included NMIs having small or partial corrosion craters in the metal matrix only around some phases of complex inclusions (*KD_cr_* = 1.3–1.9); (iv) Type IV inclusions were inactive NMIs, which barely had any corrosion craters (*KD_cr_* = 1.0–1.2) or showed any tendency for dissolution of the metal matrix around the inclusions. The SEM images of various corrosion dissolutions of metal matrix around different types of non-metallic inclusions are shown in Figure 4.

**Figure 5:**
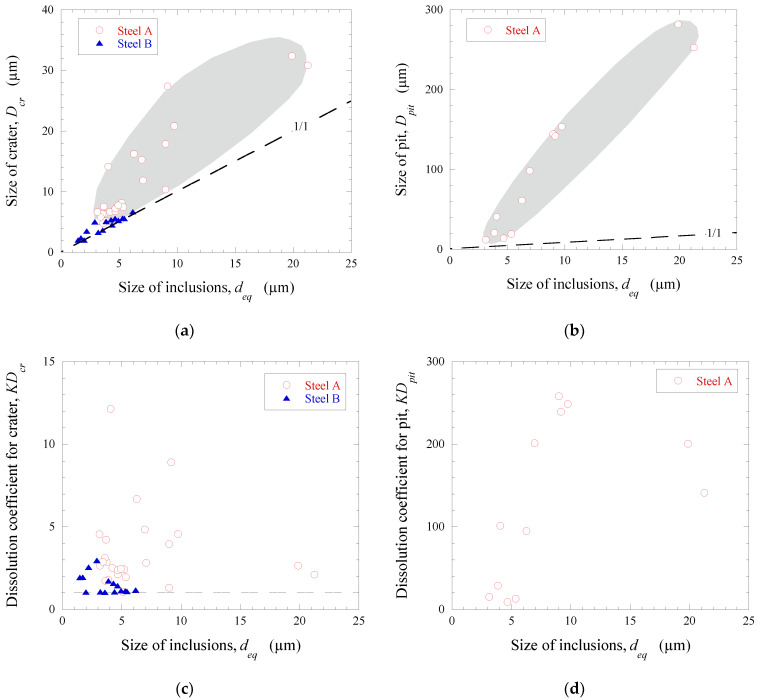
Equivalent sizes of craters (*D_cr_*) (**a**) and pits (*D_pit_*) (**b**) and coefficients of corrosion dissolution of metal matrix for craters (*KD_cr_*) (**c**) and pits (*KD_pit_*) (**d**) located around different non-metallic inclusions in Steels A and B.

The size of pits and craters around the investigated inclusions were drastically larger than the small voids (or micro-crevices) which were observed around some inclusions present on polished steel samples before chemical extraction. The 10%AA electrolyte contained methanol, and is a soft electrolyte which does not effectively dissolve the inclusions to be investigated [29]. Therefore, it was assumed that the observed craters around inclusions were a result of the dissolution of weakened zones of the metal matrix during the ChE process. Given this, the diameters of corrosion craters (*D_cr_*) and pits (*D_pit_*), and the relative coefficients of metal dissolution (*KD*), around different inclusions were used for quantitative evaluation of the influence of inclusions on the initial corrosion destruction of steel matrix. Figure 5 shows the equivalent size of craters (*D_cr_*) and pits (*D_pit_*), as well as the coefficients of corrosion dissolution of the metal matrix, for craters (*KD_cr_*) and pits (*KD_pit_*) around various non-metallic inclusions present in Steels A and B. The “1/1” line in Figure 5a,b corresponds to the inactive inclusions (Type IV) which did not contain corrosion craters and pits. The main quantitative parameters of the initial corrosion dissolution of the metal matrix around the observed non-metallic inclusions in Steels A and B after chemical extraction are given in Table 4.

It can be seen in Figure 5 and Table 4 that Steel A contained a larger number of NMIs, which had much larger diameters of craters relative to the inclusion size (*KD_cr_* = 2–12). However, the inclusions in Steel B only contained relatively small craters (*KD_cr_* = 1–3). Some inclusions in Steel A had large pits (*D_pit_* = 12–282 µm), which corresponded to the values of *KD_pit_* = 19–258, although no inclusions with pits were observed in Steel B. Moreover, it was found that the equivalent diameter of craters (*D_cr_*) and pits (*D_pit_*) in Steel A increased significantly with increased inclusion size (*d_eq_*).

The values of *D_cr_* and *KD_cr_* varied significantly for the same inclusion size, as shown in Figure 5a,c. For instance, the inclusions with *d_eq_* values between 9–10 µm had *D_cr_* values of 10–28 µm and *KD_cr_* values of 1.3–8.9. This implies that the composition of these inclusions or their phases can have a large effect on the corrosion process. Therefore, the initial corrosion dissolution of the metal matrix around various non-metallic inclusions was evaluated based on their compositions. The composition and concentration distributions of the main elements in inclusions having different corrosion dissolution effects on the steel matrix are given in Table 5. It was found that the TiN inclusions of Group 4 (see Table 3) and CaO-Al_2_O_3_-MgO oxides in complex inclusions belonging to Groups 3 and 2 did not usually contain craters of the metal matrix in both steel samples. However, the CaS inclusions of Group 1 and CaS phases in the complex inclusions of Groups 2 and 3 initiated the corrosion dissolution of the metal matrix around them. In addition, the area of the crater around these inclusions increased with increased volume of CaS in the inclusions.

A possible reason for the different corrosion dissolution effects on the metal matrix around various inclusions is discussed in the following section.

### 3.4. Physical Properties of the Matrix/Inclusion System

The corrosion process is considered, in most previous publications, to be the result of the combined effects of different chemical and physical processes, which may occur simultaneously, as detailed below:

(1) dissolution of the metal matrix due to electrochemical processes taking place between different inclusions (or phases) and the metal;
(2) chemical dissolution of a metal matrix due to chemical reactions between inclusions with the present solution (water, oil, gas, etc.), and air;
(3) dissolution of the weakened metal matrix due to formation of depletion zones of anticorrosion alloying elements in the steel matrix around inclusions containing these alloying elements;
(4) dissolution of the metal matrix around some inclusions containing some voids (or micro-crevices), which are formed during the solidification of steel; and,
(5) dissolution of a stressed metal matrix around some inclusions due to differences in physical properties (such as thermal expansion) and mechanical properties of the metal and inclusions;

It is difficult to determine which parameter/factor may have the largest effect on the initiation of a corrosion dissolution of steel matrix around inclusions. In the present study, it was assumed that factors 1 and 2 were negligibly small due to the use of a soft methanol-based 10%AA electrolyte with a short extraction time. This assumption was based on published results [29] which indicated that inclusions containing Al2O3-MgO-SiO2-CaO, CaS and TiN did not react chemically in practice with the 10%AA electrolyte even over a long period (>2 h). Moreover, in these experimental trials, the observed inclusions in the investigated steel samples did not change in the methanol-based solution during short time trials (30 min). Factors 3 (depletion zones) and 4 (voids and micro-crevices around some inclusions) were evaluated in this study. However, as was described in Section 3.2, investigation of the heterogeneity of the steel matrix around different NMIs using EDS scanning did not detect any depletion zones of alloying elements, such as Cr, Ni, Cu and Nb. Furthermore, although less than 5% of observed inclusions contained voids (<0.3 µm in size), it is doubtful that these voids could have been the main reason for the formation of much larger and deeper crates, which were formed around some inclusions in non-stirred electrolyte over a 30 min period. According to the obtained results, it was assumed that factors 3 and 4 may have had a very limited effect on the initial corrosion process under the given experimental conditions compared to the observed corrosion dissolution of the investigated steel samples. Therefore, differences in thermal expansion and the mechanical properties of the steel and various inclusions (factor 5) are considered in this study to have been the primary factors initiating corrosion dissolution of the metal matrix around some inclusions and phases.

It is known that non-metallic inclusions contained in steel represent a collection of stress concentrators. The magnitude of the stresses depends on the type and size of the inclusion, the temperature-rate conditions of deformation, the ratio of the physical and mechanical properties of the inclusion and the matrix, as well as other factors.

The reason for the occurrence of stresses in the inclusion, as well as in the surrounding matrix, is due to the temperature gradient that exists during cooling of the metal from a quenching temperature (formation of ferrite) to room temperature. Brooksbank and Andrews [30,31] proposed a basic principle for calculating such tessellated stresses. If it is assumed that the inclusions are spherical, the radial and circumferential stresses are equal at the inclusion/steel matrix. In this case, the residual stress in the inclusion/matrix interface can be evaluated using the following equation [32]:

(5) $$\sigma_{r}=\frac{\left(\alpha_{M}-\alpha_{i}\right)\Delta T}{\frac{0.5\left(1+v_{M}\right)+\left(1+2v_{M}\right)d^{3}}{E_{M}\left(1-d^{3}\right)}+\frac{1-2v_{i}}{E_{i}}}$$

where *σ_r_* is the radial stress; *α_M_* and *α_i_* are the mean linear thermal expansion coefficients of the matrix and inclusion, respectively; *ν_M_* and *ν_i_* are the Poisson ratios of the matrix and inclusion, respectively; Δ*T* is the temperature change (set as 875 °C in this study); *E_M_* and *E_i_* are the elastic Young’s modulus of the matrix and inclusion, respectively; and *d* is the volume fraction of inclusion, which was determined as follows [32]:

(6) $$d=\frac{R_{i}}{R_{M}}$$

where *R_M_* and *R_i_* are the radiuses of the matrix and inclusion, respectively.

All inclusions observed in Steels A and B contained the following phase components: Al_2_O_3_·MgO, CaO·Al_2_O_3_, CaO·2Al_2_O_3_, CaS and TiN. The basic physical properties of the various compounds and the steel matrix and the residual radial stresses (*σ_r_*) in the inclusion/matrix interface (calculated for inclusions of radius 2 µm) are listed in Table 6. As can be seen, the coefficient of linear expansion of the CaS (14.7 × 10^−6^) was significantly larger than that of the steel matrix. This implies that the CaS inclusion (or component of complex inclusion) will shrink faster during cooling compared to the steel matrix. As a result, some tensile stresses can appear in the layer of the steel matrix at a boundary with this inclusion. In this case, the calculated *σ_r_* value for CaS was negative. If the tensile stress reaches a critical value, it can promote stratification and formation of a micro-cavity (or a micro-crevice) between an inclusion and the surrounding steel matrix. Moreover, the steel matrix layer enclosed by the inclusion surface will be weaker due to increased distance between the atoms in the steel matrix. As a result, the steel matrix can be dissolved more easily and rapidly dissolved around CaS inclusions (or the CaS phase in complex inclusions) during chemical and electrolytic extractions. This agrees very well with the results obtained in the given study.

From another point of view, compounds such as the listed oxides and TiN inclusions had *α_i_* values between 5.0 and 10.0 × 10^−6^, which were lower than that for the steel matrix (12.5 × 10^−6^). In this case, the calculated *σ_r_* values for the oxides and TiN inclusions were positive. Therefore, these inclusions will shrink slowly during cooling compared to the steel matrix, which creates some resistance of inclusions to the compressive stress created in the steel layer in contact with the inclusion surface. This can lead to a compaction of the steel structure of the matrix layer. As a result, dissolution of the steel matrix around that inclusion will be difficult due to the decreased distance between the metal atoms in the surrounding layer of the steel matrix. This means that this inclusion will not promote corrosion of the surrounding area of the steel matrix. The results obtained in this study confirm that the CaO-Al_2_O_3_-MgO oxides present, and the TiN inclusions did not cause the initial corrosion of the steel matrix around these inclusions.

**Figure 6:**
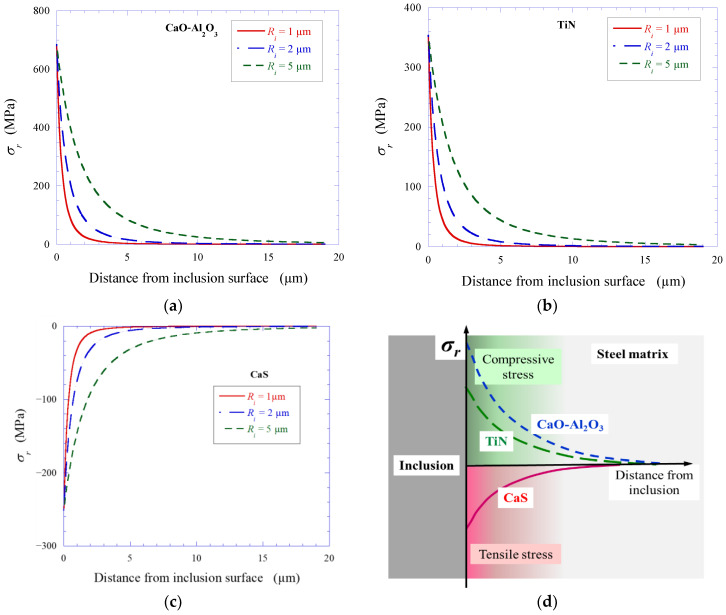
Calculated tessellated (radial) stresses around CaO·Al_2_O_3_ (**a**), TiN (**b**) and CaS (**c**) inclusions having different sizes, and a schematic illustration of the compressive and tensile stresses around different NMIs in the surrounding layer of the steel matrix (**d**).

It was found (Figure 5a) that the size of craters around corrosion-active inclusions (*D_cr_*) increased considerably with increased size of the inclusions (*d_eq_*). Therefore, the tessellated stresses in the steel matrix (*σ_r_*) generated by the CaO·Al_2_O_3_, TiN and CaS inclusions were also calculated based on inclusion size and distance from the inclusion surface. The results are shown in Figure 6a,c. The tessellated stresses at the inclusion/steel matrix depend not only on the morphologies and compositions of inclusions and their physical properties (i.e., linear thermal expansion coefficient, elastic modulus, Poisson ratio), but also on the size of the inclusion. With increase in the size of the inclusions, the calculated residual stress acting on the boundary of the inclusion/steel matrix increases. This agrees well with the obtained experimental results of this study.

During cooling of steel after hot rolling, significant compressive or tensile stresses in the adjoining layer of the steel matrix are created depending on the physical properties and sizes of non-metallic inclusions present. As shown schematically in Figure 6d, tensile stresses in the steel layer appeared around CaS inclusions, which promoted easier initial corrosion of the steel around these inclusions. However, the compressive stresses in the steel layer, which appeared around the CaO-Al_2_O_3_ and TiN inclusions, did not promote initial corrosion of the steel around these inclusions.

## 4. Conclusions

In this study, electrolytic extraction and short chemical extraction using a soft 10%AA electrolyte were applied for three-dimensional investigation of different non-metallic inclusions (varying in morphology, composition, sizes and number), and for quantitative evaluation of their effect on the initial corrosion of steel matrix around these inclusions in industrial pipeline steel samples. The main results can be summarized as follows:

1. Four groups of typical inclusions were observed in the industrial steel samples: (i) pure CaS inclusions or oxide cores completely covered by a CaS layer (97–100% of the CaS being present in the outer inclusion layer); (ii) CaO-Al_2_O_3_-MgO inclusions partially covered by a CaS layer; (iii) oxide inclusions containing small precipitations of CaS and TiN inclusions; and (iv) oxide inclusions completely covered by TiN or pure TiN inclusions.
2. The developed and tested method of short chemical extraction using a soft 10%AA electrolyte can successfully be applied for the evaluation of initial corrosion processes of the steel matrix around various non-metallic inclusions present in industrial pipeline steel samples. Quantitative parameters (such as the diameters of corrosion craters (*D_cr_*) and pits (*D_pit_*) and the relative coefficients of metal matrix dissolution (*KD*) around different inclusions) can be used for the determination and comparison of “corrosion active” inclusions and their effect on the initial corrosion of the surrounding layer of the steel matrix.
3. During cooling of steels after hot rolling, significant tensile stresses appear in the steel layer surrounding CaS inclusions due to the larger thermal expansion coefficient (*α_i_*) of CaS compared to the value of steel. This promotes faster dissolution of the steel matrix around these inclusions, which corresponds to the initial corrosion stage. However, the compressive stresses in the steel layer, which appear around CaO-Al_2_O_3_ and TiN inclusions having smaller *α_i_* values, do not contribute to the initiation of corrosion of the matrix surrounding these inclusions.

## Figures and Tables

**Table 1:** Investigations of the initial stage of the origin of pitting caused by non-metallic inclusions in pipeline and low-alloyed steels.

Steel	Corrosion Test	Investigated Parameters of NMI	Pitting Formation	Ref.
X100 steel	NS4 solution (g/L): 0.483 NaHCO_3_, 0.122 KCl, 0.181 CaCl_2_·2H_2_O and 0.131 MgSO_4_·7H_2_O, near-neutral pH	Morphologies, compositions and sizes of complex inclusions, such as Al–Mg–Ca–O, Al–Si–Ca–O and Al–Si–Ca–S–O mixtures	Formation of micropits around or at an inclusion involves the dissolution at the inclusion/matrix interface and the “drop-off” of the inclusion	[7]
X80 steel	3.5% NaCl solution in H_2_O. Temperatures: 18, 35 and 65 °C	Size and chemical composition of non-metallic inclusions	Pits are mostly initiated by mechanical defects, but some pits are initiated by non-metallic inclusions	[9]
X70 steel	Near-neutral pH NS4 solution (pH = 6.8)	Morphologyand composition of complex oxide inclusions with/without CaS	The dissolution of CaS in complex oxide inclusions induces formation of pits at the inclusions	[16]
Low-alloy steel (0.03% C, 0.25% Si, 0.1% Mn, 1.2% Cr, 0.3% Ni, 0.4% Cu, 0.002% S)	Xisha atmospheric simulated solution (0.1% NaCl, 0.05% Na_2_SO_4_ and 0.05% CaCl_2_ in H_2_O)	Morphology and size of typical non-metallic inclusions: Al_2_O_3_, ZrO_2_-Ti_2_O_3_-Al_2_O_3_ and (RE)AlO_3_-(RE)_2_O_2_S-(RE)_x_S_y_	Local corrosion by the dissolution of adjacent matrix with high energy and electrochemical activity around NMIs (Al_2_O_3_, ZrO_2_-Ti_2_O_3_-Al_2_O_3_)	[17]
Q460NH steel	0.1% NaCl, 0.05% Na_2_SO_4_ and 0.05% CaCl_2_ in H_2_O. Times: 5, 30 min and 72 h	Morphology of Al_2_O_3_ inclusions after being immersed for different times	Localized corrosion was induced at the interface between Al_2_O_3_ inclusions and the steel matrix	[18]
EH36 steel	0.5% NaCl solution in H_2_O. Times: 1, 5, 15, 60 min and 24 h	Morphology of (Ca, Mg, Al)-O_x_-S_y_ complex inclusions	Dissolution of the steel matrix leads to the formation of a micro-crevice	[22]
X80 steel	3.5% NaCl solution in H_2_O. Times: 10, 30 and 60 min	Morphologies and element distributions of typical Mg-YS composite inclusions	Steel matrix dissolves prior to Mg-YS inclusion during the initial immersion stage	[25]
Low-alloy steel (0.045%C, 0.24%Si, 0.12% Mn, 1.22% Cr, 0.32% Ni, 0.43% Cu, 0.002% S, 0.036% RE)	0.1% NaCl, 0.05% Na_2_SO_4_ and 0.05% CaCl_2_ in H_2_O (pH = 4.9)	Morphology and composition of (RE)_2_O_2_S-(RE)_x_S_y_ inclusions	Localized corrosion was initiated by the dissolution of (RE)_2_O_2_S-(RE)_x_S_y_	[26]

**Table 2:** Chemical composition of experimental steel samples used in this study (wt%).

Steel	C	Si	Mn	Cu	Cr	Ni	Ti	Al	Ca	S	N
A	0.06	0.24	0.63	0.33	0.43	0.17	0.019	0.025	0.0020	0.002	0.005
B	0.05	0.23	0.67	0.35	0.43	0.18	0.021	0.022	0.0014	0.001	0.007

**Table 3:** Typical NMI observed on film filters after electrolytic extraction and on the surface of steel samples after chemical extraction.

NMI *	SEM Image on Film Filter	SEM Image on Metal Surface	Composition (mass.%)	Size (µm)
CaS	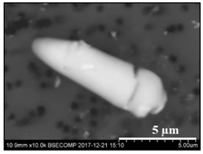	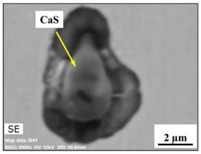	CaS—97–100%, Al_2_O_3_—0–2%, MgO—0–1%, CaO—0–2%	2.5–8.7
CAM + CaS	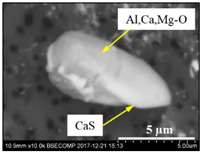	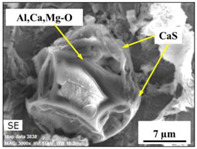	CaO—9–69%, Al_2_O_3_—2–54%, MgO—0–22%, SiO_2_—0–7%, CaS—9–62%	0.9–21.3
CAM + CaS + TiN	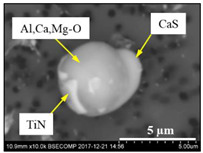	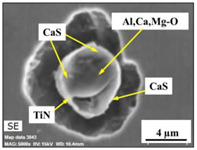	CaO—4–45%, Al_2_O_3_—4–45%, MgO—0–19%, SiO_2_—1–5%, CaS—0–50%, TiN—1–53%	1.1–5.5
TiN + CAM + CaS	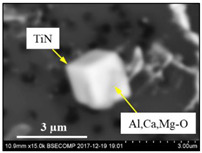	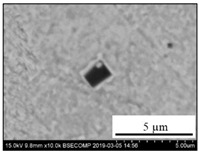	TiN—78–100%, Al_2_O_3_—0–5%, MgO—0–5%, CaO—0–5%, CaS—0–13%	0.7–4.3

* A—Al_2_O_3_; M—MgO; Si—SiO_2_; C—CaO.

**Table 4:** Main parameters of initial corrosion dissolution of metal matrix around observed non-metallic inclusions in Steels A and B after chemical extraction.

Steel	Type of NMI	*d_eq_* (µm)	*D_cr_* (µm)	*KD_cr_*	*D_pit_* (µm)	*KD_pit_*
A	I	8.6 ± 6.0 (3.1~21.3)	16.8 ± 9.5 (5.2~32.4)	4.7 ± 3.2 (1.8~12.1)	103.5 ± 93.3 (12.1~281.8)	129.3 ± 98.4 (19.1~258.1)
	II	4.4 ± 1.2 (3.2~7.1)	7.3 ± 1.9 (5.2~11.9)	2.8 ± 0.6 (2.1~4.2)		
	III	6.3 ± 3.8 (3.6~9.0)	7.6 ± 4.0 (4.8~10.4)	1.5 ± 0.3 (1.3~1.7)		
	IV	5.1	5.3	1.1		
B	I	no				
	II	2.3 ± 0.6 (1.7~2.9)	3.7 ± 1.3 (2.4~5.0)	2.5 ± 0.5 (2.0~3.0)		
	III	3.6 ± 1.4 (1.5~4.7)	4.5 ± 1.7 (2.1~5.6)	1.7 ± 0.2 (1.5~1.9)		
	IV	4.1 ± 1.3 (2.0~5.5)	4.3 ± 1.4 (2.1~5.6)	1.1 ± 0.1 (1.0~1.2)		

(~)—are the minimum and maximum values of parameter.

**Table 5:** Composition and concentration distributions of the main elements in inclusions having different corrosion dissolutions effects on the steel matrix.

NMI	SEM Image	Concentration Mapping
Type I	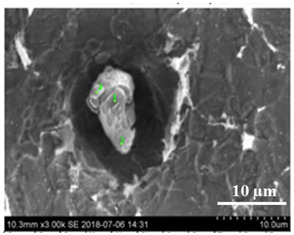	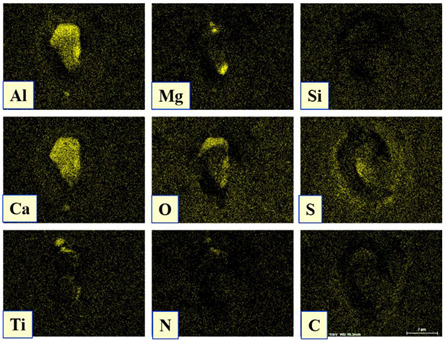
Type II	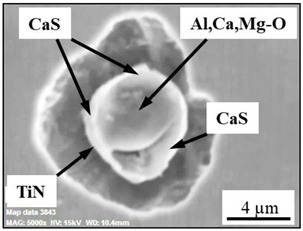	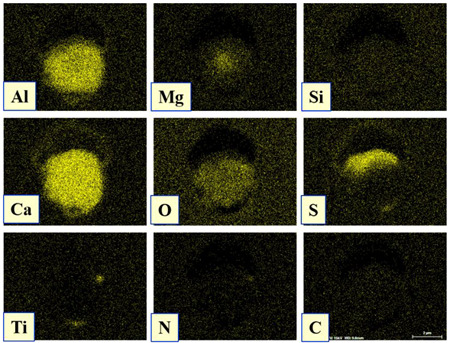
Type III	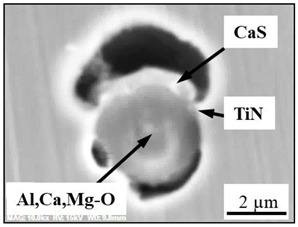	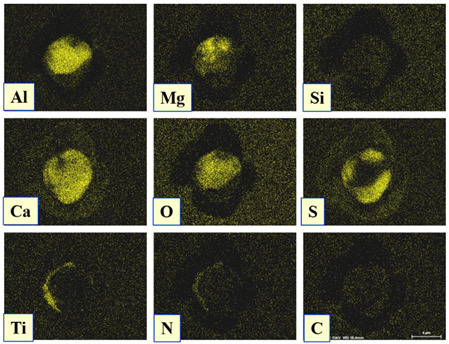
Type IV	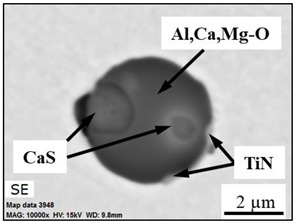	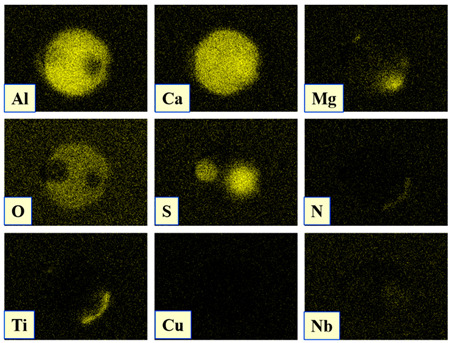

**Table 6:** Physical properties of different inclusions (or components in complex inclusions) and the steel matrix [31].

Compounds	Poisson’s Ratio, *v_i_*	Young’s Modulus, *E_i_* (GPa at 20 °C)	Thermal Expansion Coefficient, *αi* (×10^−6^/°C)	Residual Radial Stress, *σr* * (MPa)
CaS	0.300	56.7	14.7	−250.8
3CaO·Al_2_O_3_	0.234	115	10.0	285.0
12CaO·7Al_2_O_3_	0.234	115	7.8	684.0
CaO·Al_2_O_3_	0.234	115	6.5	535.8
CaO·2Al_2_O_3_	0.234	115	5.0	855.0
Al_2_O_3_	0.250	402	8.0	513.0
Al_2_O_3_·MgO	0.260	271	8.4	467.4
TiN	0.192	323	9.4	353.4
Steel matrix	0.290	206	12.5	-

* *σ_r_* values calculated for inclusions with *R_i_* = 2 µm and *R_m_* = 79 µm.

## References

[1] Shibaeva T.V., Laurinavichyute V.K., Tsirlina G.A., Arsenkin A.M., Grigorovich K.V. (2014). The effect of microstructure and non-metallic inclusions on corrosion behavior of low carbon steel in chloride containing solutions. Corros. Sci..

[2] Weber R.A., Somers B.R., Kaufmann E.J. (1996). Low-Carbon, Age-Hardenable Steels for Use in Construction: A Review.

[3] Edmonds D.V., Cochrane R.C. (2005). The effect of alloying on the resistance of carbon steel for oilfield applications to CO_2_ corrosion. Materials Research-ibero-american. J. Mater..

[4] Wu W., Liu Z., Wang Q., Li X. (2020). Improving the resistance of high-strength steel to SCC in a SO_2_-polluted marine atmosphere through Nb and Sb microalloying. Corros. Sci..

[5] Dong J., Li C., Liu C., Huang Y., Yu L., Li H., Liu Y. (2017). Microstructural and mechanical properties development during quenching-partitioning-tempering process of Nb-V-Ti microalloyed ultra-high strength steel. Mater. Sci. Eng. A.

[6] Xie Y., Xu L., Gao C., Chang W., Lu M. (2012). Corrosion behavior of novel 3% Cr pipeline steel in CO_2_ Top-of-Line Corrosion environment. Mater. Des..

[7] Li Y., Liu J., Deng Y., Han X., Hu W., Zhong C. (2016). Ex situ characterization of metallurgical inclusions in X100 pipeline steel before and after immersion in a neutral pH bicarbonate solution. J. Alloys Compd..

[8] Williams D.E., Kilburn M.R., Cliff J., Waterhouse G.I.N. (2010). Composition changes around sulphide inclusions in stainless steels, and implications for the initiation of pitting corrosion. Corros. Sci..

[9] Wang Y., Cheng G., Li Y. (2016). Observation of the pitting corrosion and uniform corrosion for X80 steel in 3.5 wt.% NaCl solutions using in-situ and 3-D measuring microscope. Corros. Sci..

[10] Liu C., Revilla R.I., Liu Z., Zhang D., Li X., Terryn H. (2017). Effect of inclusions modified by rare earth elements (Ce, La) on localized marine corrosion in Q460NH weathering steel. Corros. Sci..

[11] Li J., Gao X., Du L., Liu Z. (2017). Relationship between microstructure and hydrogen induced cracking behavior in a low alloy pipeline steel. J. Mater. Sci. Technol..

[12] Torkkeli J., Saukkonen T., Hänninen H. (2015). Effect of MnS inclusion dissolution on carbon steel stress corrosion cracking in fuel-grade ethanol. Corros. Sci..

[13] Wranglen G. (1974). Pitting and sulphide inclusions in steel. Corros. Sci..

[14] Wei J., Dong J., Ke W., He X. (2015). Influence of inclusions on early corrosion development of ultra-low carbon bainitic steel in NaCl solution. Corrosion.

[15] Anijdan S., Mousavi H., Arab G., Sabzi M., Sadeghi M., Eivani A.R., Jafarian H.R. (2021). Sensitivity to hydrogen induced cracking, and corrosion performance of an API X65 pipeline steel in H_2_S containing environment: Influence of heat treatment and its subsequent microstructural changes. J. Mater. Res. Technol..

[16] Wang L., Xin J., Cheng L., Zhao K., Sun B., Li J., Wang X., Cui Z. (2019). Influence of inclusions on initiation of pitting corrosion and stress corrosion cracking of X70 steel in near-neutral pH environment. Corros. Sci..

[17] Liu C., Li X., Revilla R.I., Sun T., Zhao J., Zhang D., Yang S., Liu Z., Cheng X., Terryn H. (2021). Towards a better understanding of localised corrosion induced by typical non-metallic inclusions in low-alloy steels. Corros. Sci..

[18] Liu C., Revilla R.I., Zhang D., Liu Z., Lutz A., Zhang F., Zhao T., Ma H., Li X., Terryn H. (2018). Role of Al_2_O_3_ inclusions on the localized corrosion of Q460NH weathering steel in marine environment. Corros. Sci..

[19] Jin T.Y., Liu Z.Y., Cheng Y.F. (2010). Effect of non-metallic inclusions on hydrogen-induced cracking of API5L X100 steel. Int. J. Hydrog. Energy.

[20] Szklarska-śmialowska Z., Lunarska E. (2004). The effect of sulfide inclusions on the susceptibility of steels to pitting, stress corrosion cracking and hydrogen embrittlement. Mater. Corros..

[21] Reformatskaya I.I., Podobaev A.N., Florianovich G.M., Ashcheulova I.I. (1999). Evaluation of the corrosion resistance of low-carbon pipe steels under conditions of hot-water supply. Prot. Met..

[22] Wang Y., Zhang X., Cheng L., Liu J., Hou T., Wu K. (2021). Correlation between active/inactive (Ca, Mg, Al)-Ox-Sy inclusions and localised marine corrosion of EH36 steels. J. Mater. Res. Technol..

[23] Serov G.V., Komissarov A., Tikhonov S., Sidorova E.P., Kushnerev V., Mishnev P.A., Kuznetsov D.V. (2019). Effect of Deoxidation on Low-Alloy Steel Nonmetallic Inclusion Composition. Refract. Ind. Ceram..

[24] Liu P., Zhang Q., Li X., Hu J., Cao F. (2021). Insight into the triggering effect of (Al, Mg, Ca, Mn)-oxy-sulfide inclusions on localized corrosion of weathering steel. J. Mater. Sci. Technol..

[25] Hou Y., Li T., Li G., Cheng C. (2020). Mechanism of Yttrium composite inclusions on the localized corrosion of pipeline steels in NaCl solution. Micron.

[26] Liu C., Jiang Z., Zhao J., Cheng X., Liu Z., Zhang D., Li X. (2020). Influence of rare earth metals on mechanisms of localised corrosion induced by inclusions in Zr-Ti deoxidised low alloy steel. Corros. Sci..

[27] Sidorova E., Karasev A.V., Kuznetsov D., Jönsson P.G. (2019). Modification of non-metallic inclusions in oil-pipeline steels by ca-treatment. Metals.

[28] Karasev A.V., Suito H. (1999). Analysis of Size Distributions of Primary Oxide Inclusions in Fe-10 mass pct Ni-M (M = Si, Ti, Al, Zr, and Ce) Alloy. Metall. Mater. Trans. B.

[29] Inoue R., Kiyokawa K., Tomoda K., Ueda S., Ariyama T. Three-Dimensional Estimation of Multi-Component Inclusion Particle in Steel. Proceedings of the 8th International Workshop on Progress in Analytical Chemistry and Materials Characterization in the Steel and Metal Industries (CETAS-2011).

[30] Brooksbank D., Andrews K. (1968). Thermal expansion of some inclusions found in steels and relation to tessellated stresses. J. Iron Steel Inst..

[31] Brooksbank D., Andrews K. (1972). Stress fields around inclusions and their relation to mechanical properties. Int. Conf. Prod. Appl. Clean Steels.

[32] Brooksbank D., Andrews K. (1969). Tessellated stresses associated with some inclusions in steel. J. Iron. Steel Inst..

